# Long-Term Social Human-Robot Interaction for Neurorehabilitation: Robots as a Tool to Support Gait Therapy in the Pandemic

**DOI:** 10.3389/fnbot.2021.612034

**Published:** 2021-02-23

**Authors:** Nathalia Céspedes, Denniss Raigoso, Marcela Múnera, Carlos A. Cifuentes

**Affiliations:** Departament of Biomedical Engineering, Colombian School of Engineering Julio Garavito, Bogotá, Colombia

**Keywords:** COVID-19, gait rehabilitation, Lokomat, long-term human-robot interaction, biofeedback, socially assistive robotics

## Abstract

COVID-19 pandemic has affected the population worldwide, evidencing new challenges and opportunities for several kinds of emergent and existing technologies. Social Assistive Robotics could be a potential tool to support clinical care areas, promoting physical distancing, and reducing the contagion rate. In this context, this paper presents a long-term evaluation of a social robotic platform for gait neurorehabilitation. The robot's primary roles are monitoring physiological progress and promoting social interaction with human distancing during the sessions. A clinical validation with ten patients during 15 sessions were conducted in a rehabilitation center located in Colombia. Results showed that the robot's support improves the patients' physiological progress by reducing their unhealthy spinal posture time, with positive acceptance. 65% of patients described the platform as helpful and secure. Regarding the robot's role within the therapy, the health care staff agreed (>95%) that this tool can promote physical distancing and it is highly useful to support neurorehabilitation throughout the pandemic. These outcomes suggest the benefits of this tool to be further implemented in the pandemic.

## 1. Introduction

In light of the rapid spread of COVID-19, several healthcare services are looking for strategies to promote physical distancing and enhance healthcare procedures. Physical distancing and isolation measures are adopted worldwide (WHO, 2020). Studies highlight the importance of these actions to decrease the transmission rate (Jarvis et al., 2020), reduce the peak incidence, delay the epidemic (Zhang et al., 2020), and minimize the intrahospital interactions (Aymerich-Franch, 2020). For instance, there is a concern to seek adaptive strategies to continue offering neurorehabilitation services during the COVID-19 pandemic, as the people with disabilities and chronic progressive diseases require constant monitoring and care (Leocani et al., 2020; Russo and Trabacca, 2020). the exploration of new technologies to support the general population's health is studied (Sakel et al., 2020).

In this context, Social Assistive Robotics (SAR) can play a critical role in real environments, mainly to promote physical distancing and support the rehabilitation's continuity. SAR shares with Assistive Robotics (AR), not only the goal of providing physical assistance to patients, but also to aid users through cognitive support, and social interaction. Thus, social robots need to perform high degree autonomy tasks to achieve natural interaction (Duffy et al., 1999; Feil-Seifer and Mataric, 2011). SAR based applications have been developed in multiple clinics (Cifuentes et al., 2020), home-based (Campa and Campa, 2016), and educational (Heerink et al., 2016) areas. The outcomes of these studies show positive effects regarding the motivation (Winkle et al., 2018), adherence to medical treatments (Fasola and Matarić, 2010; Heerink et al., 2016), social interaction (Agrigoroaie and Tapus, 2016), among others. Within the COVID-19 pandemic, several researchers highlight the use of SAR through two main tasks: (i) monitoring the patients, and (ii) connecting doctors (who are exposed to a high risk of contagion) with patients using teleoperation (Aymerich-Franch, 2020; Hollander and Carr, 2020). Scassellati and Vázquez (2020) proposed using SAR to sustain social distancing and serve as health monitoring tools in high-risk areas. Khaleghi et al. (2020) remarked on social robots' opportunities to provide services remotely and aid the healthcare staff. Furthermore, some studies proposed SAR to interact in hospital environments and deal with mental health and well-being (Tavakoli et al., 2020).

In this study, a social robotic platform for neurorehabilitation with Lokomat for during and after the COVID-19 pandemic is presented. Lokomat is a device that combines a bodyweight support system and a robotic orthosis to assist the gait using repetitive specific tasks and the principle of neuroplasticity (Swinnen et al., 2017). This platform allows the measurement of different parameters: the patients' strength, mechanical stiffness, and the range of motion during the walking. These parameters enable the physiotherapist to straighten the therapy according to the objective of each patient (Gittler M, 2018). However, some parameters not detected by the Lokomat are essential during the rehabilitation (e.g., heart rate, the patient's posture, and the patient's fatigue level). In this sense, clinicians measure those parameters directly using external equipment (heart rate), visually (posture), and asking the patient (level of fatigue) verbally. Monitoring the heart rate enables the observation of the physical progress in terms of cardiovascular functioning, and correcting the spinal posture to maintain it healthy, promotes back health, allows muscles to work correctly, and decrease muscle fatigue (Sante, 2012; Daroff, 2016; Weaver and Ferg, 2020). Thus, SAR can be a complementary tool to automatize these parameters, provide feedback, interact with the patients during the therapy, and promote physical distancing.

This paper presents the long-term evaluation of a social robotic platform in neurorehabilitation with Lokomat. The patients performed a repeated measures study (due to the heterogeneity of the pathologies) during 15 sessions, where two conditions were established (i.e., control and robot-assisted therapy). The robot's primary roles were to assist the patient through physiological parameter feedback (e.g., posture correction and heart rate and perceived exertion monitoring) and motivational approaches. Furthermore, the platform's assessment seeks to observe the patient's progress through the therapies and their perception toward the robot. This platform can represent an opportunity to provide rehabilitation safely during and after the COVID-19 pandemic.

This paper is organized as follows. Section 2 presents the related work of social robotic platforms implemented in healthcare and rehabilitation areas. Section 3 describes the social robotic platform and the assessment methods used to evaluate its functionality and effectiveness in a neurorehabilitation scenario. Section 4 introduces the long-term results observed during the session regarding the physiological parameters and the patients' perception of social robots. Finally, the results and conclusions are presented at the end of this paper.

## 2. Related Work

Although robot-assisted therapies as Lokomat are successful, cognitive approaches to enhance the treatment are also essential to provide care and physical assistance. SAR is currently being used in different areas (Yang et al., 2015; Heerink et al., 2016; Peleka et al., 2018). In healthcare, several studies are focused on measure the effects of social robots during rehabilitation procedures in terms of adherence to the treatments, assistance and perception (Matarić et al., 2007; Casas et al., 2019).

Different studies show the capabilities of SAR in post-stroke patients to support rehabilitation procedures regarding the cognitive approach. Robinson et al. (2013), proposed a social care robotic platform to aid post-stroke patients through contactless assistance. The system was tested in a pilot study, where the mobile robot supports the therapy through encouragement and reminders. The researchers found that welfare robots were well-received by stroke survivors and positively impacted willingness to undergo rehabilitation plans. In Libin and Libin (2004), a social robot was designed to create a relationship with the user using extroversion and introversion techniques. The robot also offers an adaptive behavior, capable of adjusting social interaction (e.g., interaction/proxemic distances, personalized speed, and vocal content) based on the users' personality traits and task performance. The reported results provide evidence of the user's preference for the personality matching robot and its benefits over rehabilitation performance. Currently, Polak and Levy-Tzedek (2020) presented a Pepper robot aimed at supporting upperlimb rehabilitation in a long-term study. The design of SAR based therapy considered the clinician's experience and perception. The robot was capable of promoting different skills and gives the patients trust to perform the games.

In contrast, social robots can also assist patients in employing physiological parameters monitoring and providing feedback. For instance, Kozyavkin et al. (2014) use a humanoid robot to help cerebral palsy patients during motor training activities. The primary role of the robot was supporting the children. The results indicate that patients like to interact with the robot and even suggest integrating them in other rehabilitation scenarios. The outcomes also show that the social robot has a positive effect on the patients regarding their motivation and their willingness to complete the health procedures. Similarly, in pediatric rehabilitation, researchers have highlighted the potential use of robots to actively engage the children to the rehabilitation and increase the commitment to perform the exercises (Carrillo et al., 2017; Pulido et al., 2017). Martín et al. (2020) developed a physical therapy assisted by a humanoid robot to guide the patients through imitation of several postures. Depending on the patients' performance, the robot congratulates or corrects the users. The system was implemented in real environment set-ups, showing that the system was reliable and could improve the therapist's and patient's tasks during rehabilitation procedures.

Furthermore, social robots are being used in alternative rehabilitation areas. For instance, in cardiac rehabilitation, a humanoid robot was implemented to monitor and support patients with cardiovascular diseases (Casas et al., 2020). The robot gives the clinicians alerts to warn emergency events, provide motivation and correct the patient's physical activity performance. The outcomes highlight the robot's potential in this scenario and the positive effects on cardiovascular physiology.

Finally, in our previous work (Cespedes et al., 2020), the development of a SAR interface was presented. The social robot's roles were to support and encourage patients with neurological diseases during gait rehabilitation with Lokomat. These patients perform two sessions (one assisted by the robot and one conventional therapy). Preliminary findings show that patients tend to improve their posture with the use of the robot. Overall, the results regarding social robots in rehabilitation are positive. However, few studies assess SAR's effects in long-term periods, avoiding the fact that the novelty effect can decrease with time (Kasap and Magnenat-Thalmann, 2012), and social interaction could be affected. Most of these studies also integrate social robotics in conventional therapies rather than robot-assisted therapies as Lokomat rehabilitation. In this context, it is crucial to assess the effects of a complementary tool (SAR) that support rehabilitation from other approaches.

## 3. Methodology

This section describes the methodology carried out to evaluate the social robot effect during a long-term study in neurorehabilitation with Lokomat. Within the method, three steps were followed: (i) social robotic platform architecture, (ii) the experimental protocol, and (iii) data analysis.

### 3.1. Social Robotic Platform Architecture

**Figure 1:**
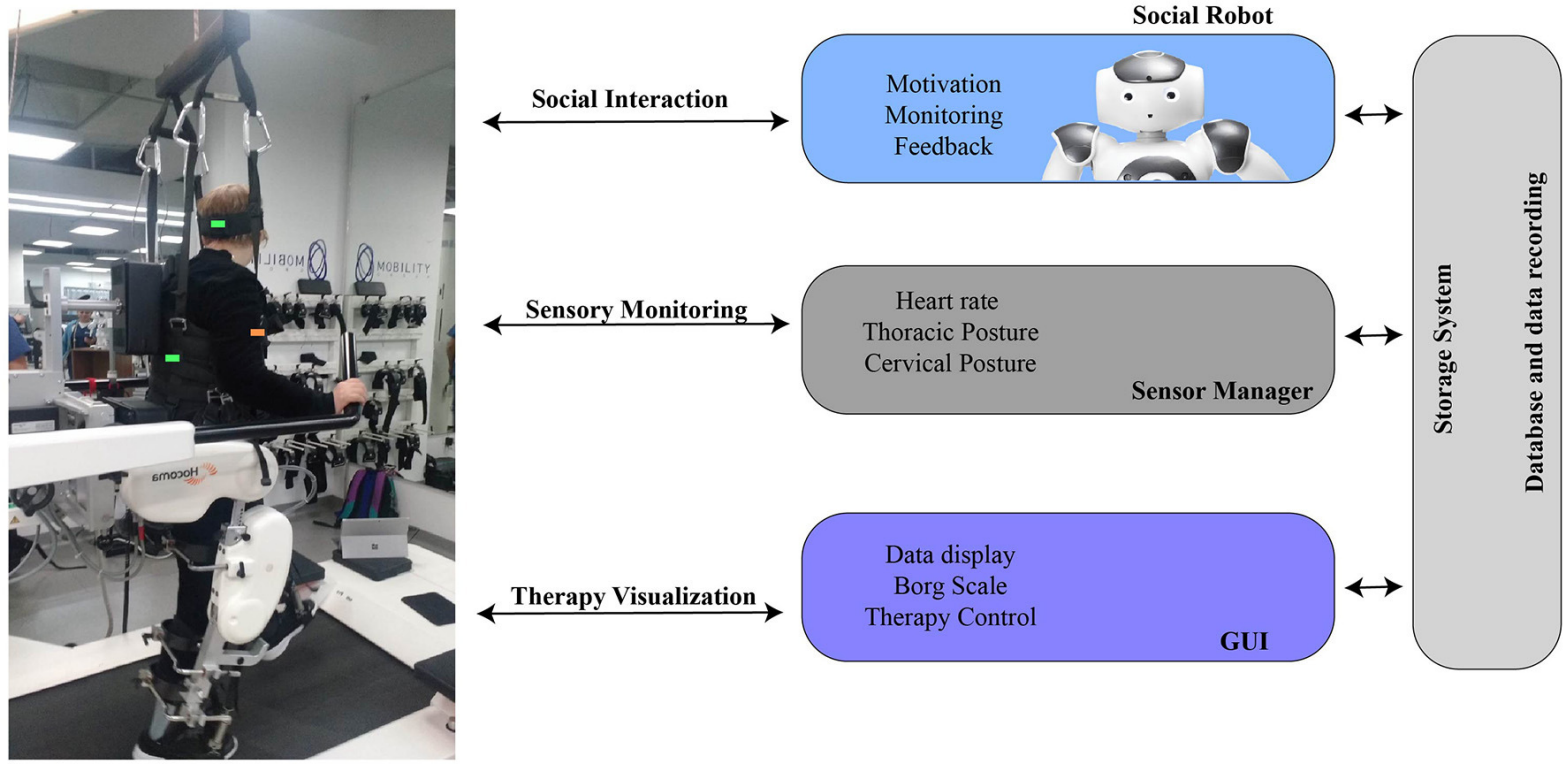
Social robotic platform architecture for neurological rehabilitation with Lokomat.

Figure 1, shows the architecture of the social robotic platform proposed for neurorehabilitation with the Lokomat Scenario. The system is composed of three main modules: (i) the sensory module, which allows the acquisition and processing of the physiological data, (ii) the social robot module, in charge of the social interaction and the assistance of the patients, and (iii) the graphical user interface used to visualize the parameters of the parameters and control the therapy flow.

***Sensory module:*** As mentioned previously in the sensory module, the physiological data are acquired and processed. The system's physiological parameters are the spinal posture (*thoracic and cervical posture*), the *heart rate*, and the *Borg scale*. The interface performs downsampling (1 Hz) to obtain simultaneous data from the sensors, then the data are stored on the database. *Cervical and Thoracic postures* are measured by an IMU BNO055 (Adafruit, USA), and inclination angles in the sagittal, coronal and traversal planes are obtained. A Zephyr HxM sensor (Medtronic, New Zealand) measures the heart rate. The sensor is located in the patient's chest to monitor cardiovascular functioning. Finally, the Borg Scale is a subjective measurement commonly used in rehabilitation to measure the patients' perceived exertion during the exercise (Compagnant et al., 2017). The robot asks the scale in a frequency of 5 min across the session. The therapist records the scale in the database.

**Figure 2:**
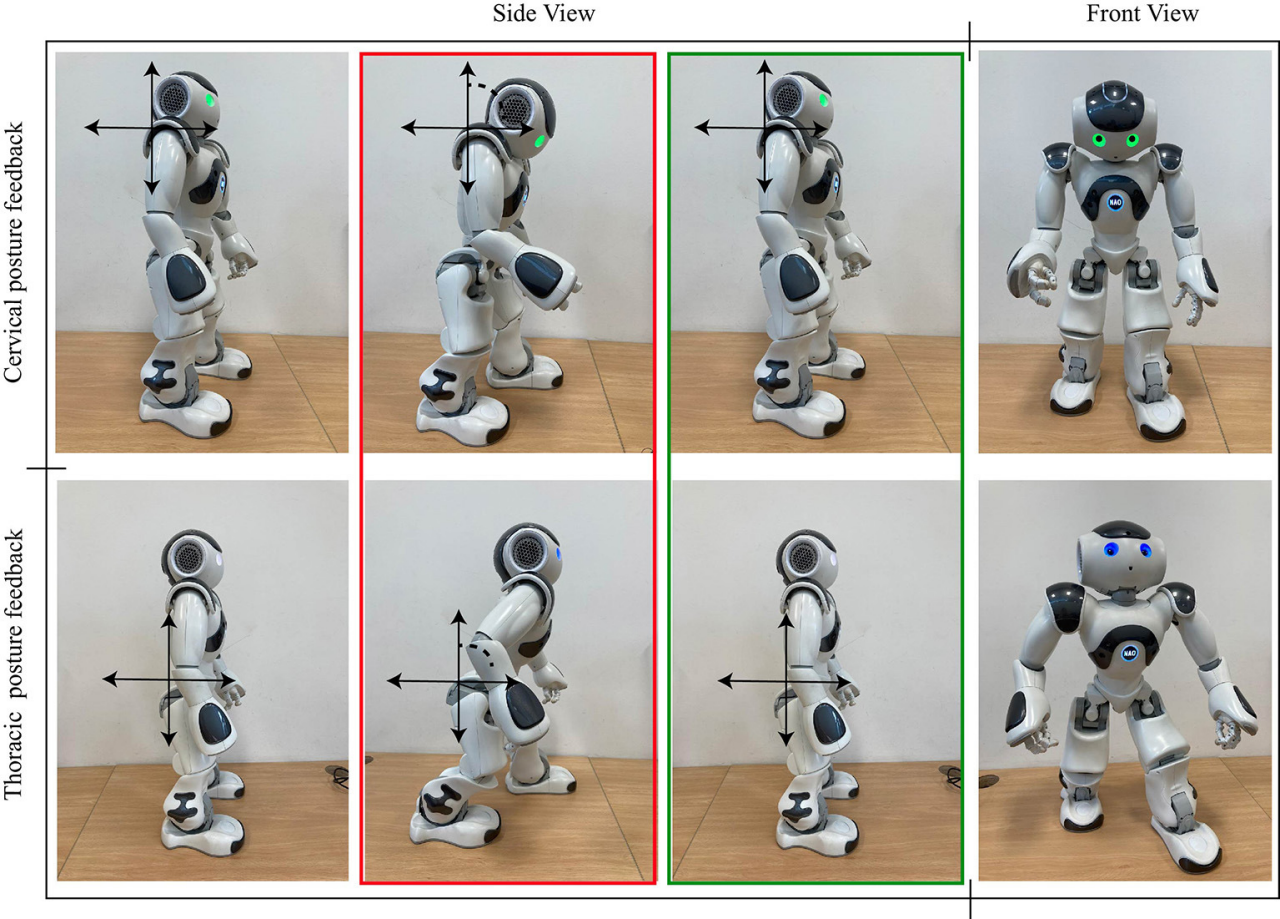
Robot's behaviors regarding the posture feedback provided to the patient during Lokomat sessions.

***Social robot module:*** A NAO V6 robot (Softbank Robotics, France) was used to achieve the interaction. The primary robot's role is to provide feedback to the patient of physiological parameters (i.e., cervical and thoracic posture, heart rate) and motivate them during therapy development. Additionally, the robot supports the therapists while they perform other tasks during the session. The robot is located in front of the patient during the exercise, guiding their performance by imitating healthy postures (Figure 2). Thus, the platform enables the physical distancing between the clinicians and the patient. The feedback given by the robot includes non-verbal and verbal gestures. Three feedback categories are proposed: (i) Heart rate feedback, provide alerts regarding the patient's high heart rate during the gait rehabilitation. (ii) Posture feedback, where the robot uses a verbal phrase to indicate the patient the performance of an unhealthy posture, and body gestures to show the patient how to correct and maintain a proper posture. This type of feedback is given to correct cervical and thoracic spinal postures. Finally, (iii) motivational feedback supports the patients through encouraging phrases. The non-verbal gestures and the conversation scheme designed for the robot is developed with a rule-based algorithm. This algorithm depends on the events triggered during the sessions and the types of feedback presented previously. For instance, the motivational phrases are performed when the patient accomplish a healthy posture. The conversation contents include a set of phrases (e.g., “*you are doing ok*,” “*We almost finished the sessions*,” “*Great!, you are improving the posture”*) that are performed randomly.

**Figure 3:**
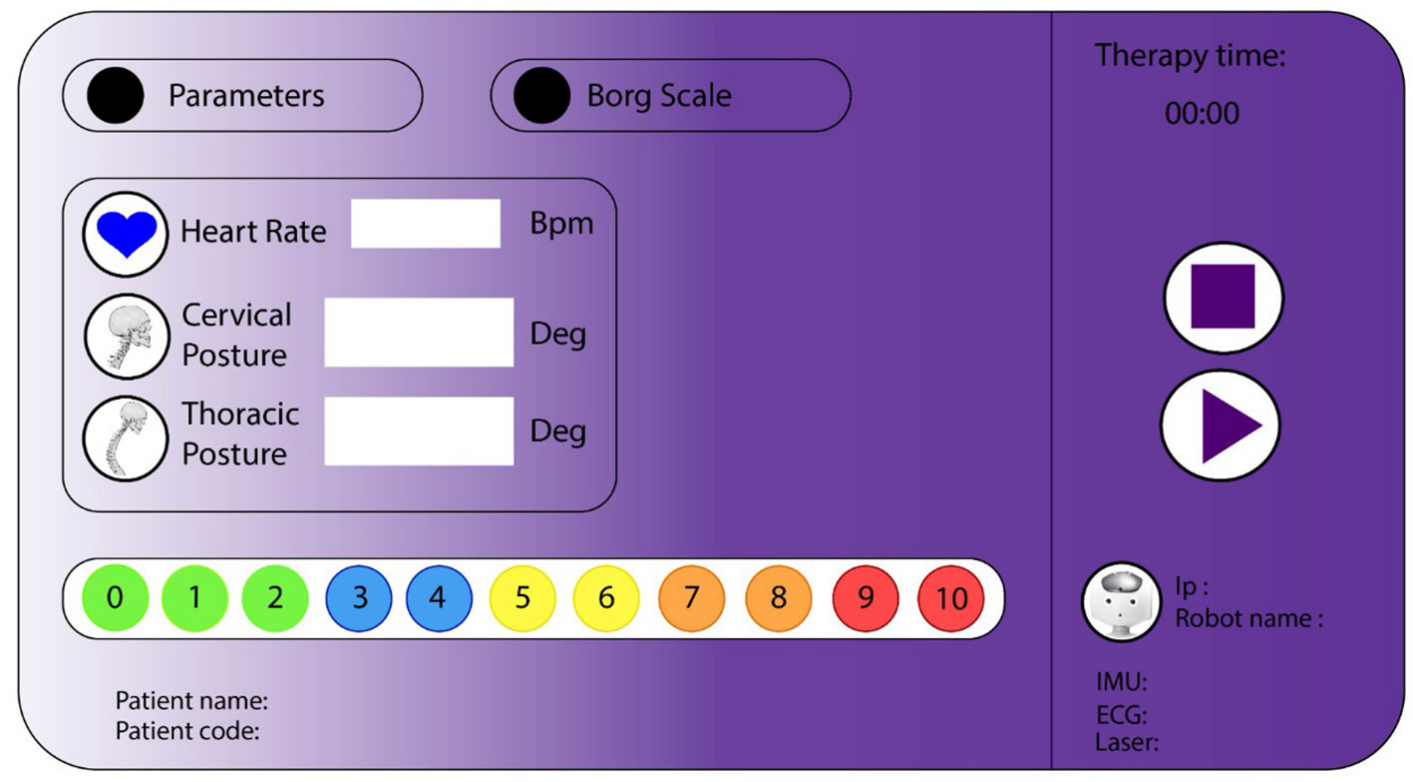
Graphical user interface for neurological rehabilitation with Lokomat. The visualization area contains labels that show physiological parameters and patients' information. The control flow area has buttons to start and stop the therapy.

***Graphical User Interface:*** This interface is in charge of visualizing the therapy's data and control the session flow (Figure 3). A tablet Surface Pro (Windows, USA) was used to display the interface. This tool also allows therapists to interact with the patient and manage the session.

### 3.2. Experimental Protocol

A total of 10 patients were recruited during the study. These patients performed actively Neurological Rehabilitation with Lokomat at Mobility Group Rehabilitation Center located in Bogota, Colombia. These patients voluntarily agreed to perform the rehabilitation assisted by the robot during 15 sessions (approx. 5 months, where 1 session was conducted per week^1^, the sessions lasted between 40 and 60 min) (Bickmore and Picard, 2005; Sabelli et al., 2011). However, within this study, only 60% of the patients finished rehabilitation with Lokomat. Table 1 shows the demographic data of the patients and their pathologies.

**Table 1:** Demographic data of the patients within the study.

	**Patients' data**	
Participants	10	
Gender	3 females	7 males
Age (years), mean ± SD	35.5 ± 9.98	
Weight (Kg), mean ± SD	71.7 ± 7.60	
Pathology (%)	Stroke (60%) Spinal cord injury (40%)	

#### 3.2.1. Experimental Design

**Figure 4:**
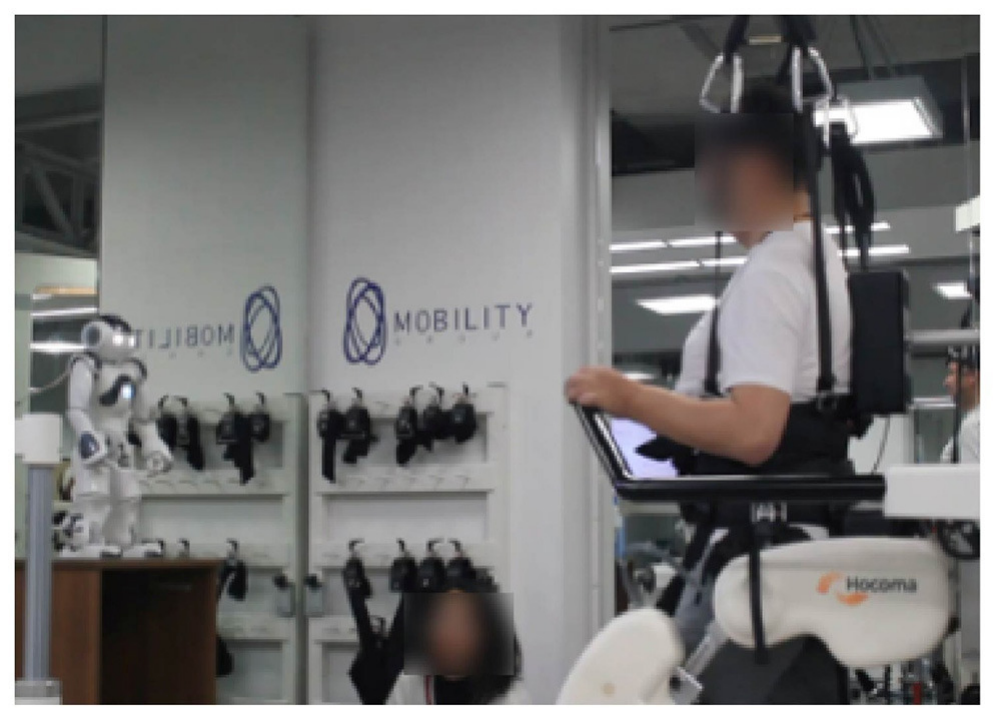
The diagram illustrates the robot condition performed in the experimental design.

**Figure 5:**
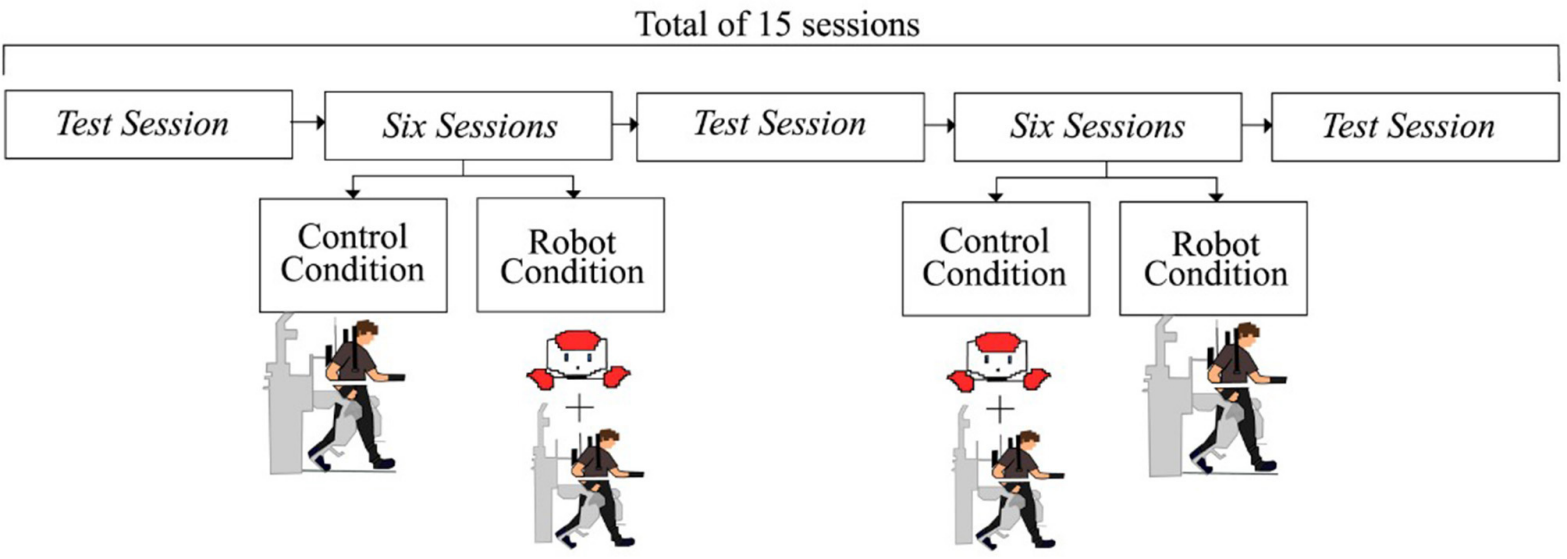
The diagram illustrates the experimental design. *Test sessions* and six therapy sessions are developed to evaluate the robot's effects over the patients.

Due to the patient's heterogeneity, a repeated measures study was performed to evaluate the patient's progress during neurological Rehabilitation with Lokomat. Two conditions were established: a control condition and a robot condition (Figure 4); during both conditions the patients also received support from the healthcare staff. Figure 5 shows the design of the study. *Test sessions* are performed at the beginning, in the middle, and at the end of the study. Within these *Test sessions*, only physiological parameters were measured and were taken as a baseline. Then, the patients were assigned randomly to start with one condition (either control or robot) during six sessions (one session per week). Finally, considering the start condition, the patients changed the scenario during another six sessions (one session per week).

***Control Condition:*** Within this condition, the participants performed a conventional session of neurological rehabilitation with Lokomat. However, to measure the physiological data and compare them to the other conditions, the patients were monitored through the sensory module. The patients received assistance and assessment from the healthcare staff.

***Robot Condition:*** Within this condition, the participants performed the sessions assisted by the social robot. As was explained in the section that describes the architecture, the robot's role was focused on providing motivational feedback and support patients' rehabilitation throughout the monitoring of physiological parameters (e.g., cervical, thoracic posture, and heart rate). Furthermore, the healthcare staff was supervising the therapy and gave additional feedback to the patient (e.g., ankle gait patterns correction).

#### 3.2.2. Experimental Criteria

***Inclusion Criteria:*** The patients considered in this study were those who actively perform neurorehabilitation therapies with Lokomat. Overall, the patients had to be able to understand and follow the robot instructions. The pathologies considered in the study were: spinal cord injury (hemiplegia, paraplegia) and stroke.

***Exclusion Criteria:*** Patients with neurodegenerative diseases such as Multiple sclerosis, Alzheimer's Parkinson's, among others, were not included in the study. Additionally, patients who had invasive electronic devices (e.g., pacemakers) cannot perform the study due to the interference that can cause the system's sensors.

### 3.3. Data Analysis

Two types of variables were analyzed to evaluate the robot assistance: on the one hand, quantitative variables included the unhealthy posture time, the Borg scale, and the heart rate at training. On the other hand, qualitative variables integrate the UTAUT questionnaire to observe the patient's perceptions of the robot's role.

***PPt [%]:*** This value describe the time during which the patient presents an unhealthy spinal posture (i.e., thoracic and cervical posture) in the Lokomat sessions. First the values considered as a healthy posture were calibrated for each patient to measure this parameter. With these values, a threshold was determined to calculate the unhealthy posture (i.e., 10 degrees over/under the threshold). Finally, the time of this event was calculated and normalized with the test sessions. Equation (1), where *PPt*_*norm*_, is the time of unhealthy spinal posture; *PPt*_*n*−*session*_ is the time of unhealthy spinal posture in the current session, and *PPt*_*test*−*session*_ is the time of unhealthy spinal posture in the test session.

(1) $$\begin{array}{l} PPt_{norm}=\frac{\left(PPt_{n-session}-PPt_{test-session}\right)}{PPt_{test-session}}*100 \end{array}$$

***Heart Rate [Bpm]:*** This parameter corresponds to the heart rate acquired during the rehabilitation. The parameter was averaged in each session.

***Borg Scale:*** This parameter corresponds to the exertion perceived during the exercise. The scale used in the rehabilitation center varies between 0 (i.e., rest) and 10 (i.e., exertion perceived as high). This value was averaged in each session.

***UTAUT questionnaire:*** A UTAUT questionnaire was applied at the end of the rehabilitation to measure the clinicians' perception and attitudes to the social robot. This measurement is based on the Almere model (Heerink et al., 2010), which evaluates the perception through different constructs: Social Presence (SP), Perceived Sociability (PS), Perceived Trust (PT), Ease of Use (EU), Safety (S), Perceived Utility (PU), and Usefulness (U). A total of 26 closed questions (answered by a Likert scale) and two open items were implemented in the questionnaire (Supplementary Table 1).

***COVID-related questionnaire:*** A short-questionnaire was implemented to the clinicians' to measure their perception toward the robot during the pandemic. For instance, the questions were related to the usability of the robot in the pandemic and how it can be a tool to support neurorehabilitation (Supplementary Table 2). The questionnaire was composed of six closed questions and three open questions.

A Wilcoxon Signed-Rank test was applied to compare the patient's progress in both conditions. The Wilcoxon Signed Rank is a non-parametric test used to compare two related samples (i.e., in this case compare the robot and control condition performed by the same patient) to assess whether their population mean rank differ (Wilcoxon, 1945). A descriptive analysis was performed for the closed questions in the qualitative parameters, and a textual data analysis test was performed for the open items.

## 4. Results

As mentioned in the methodology section, two types of variables were observed (i.e., qualitative and quantitative). This section presents the results of patients who participated in the study during 15 sessions of neurorehabilitation with Lokomat.

**Figure 6:**
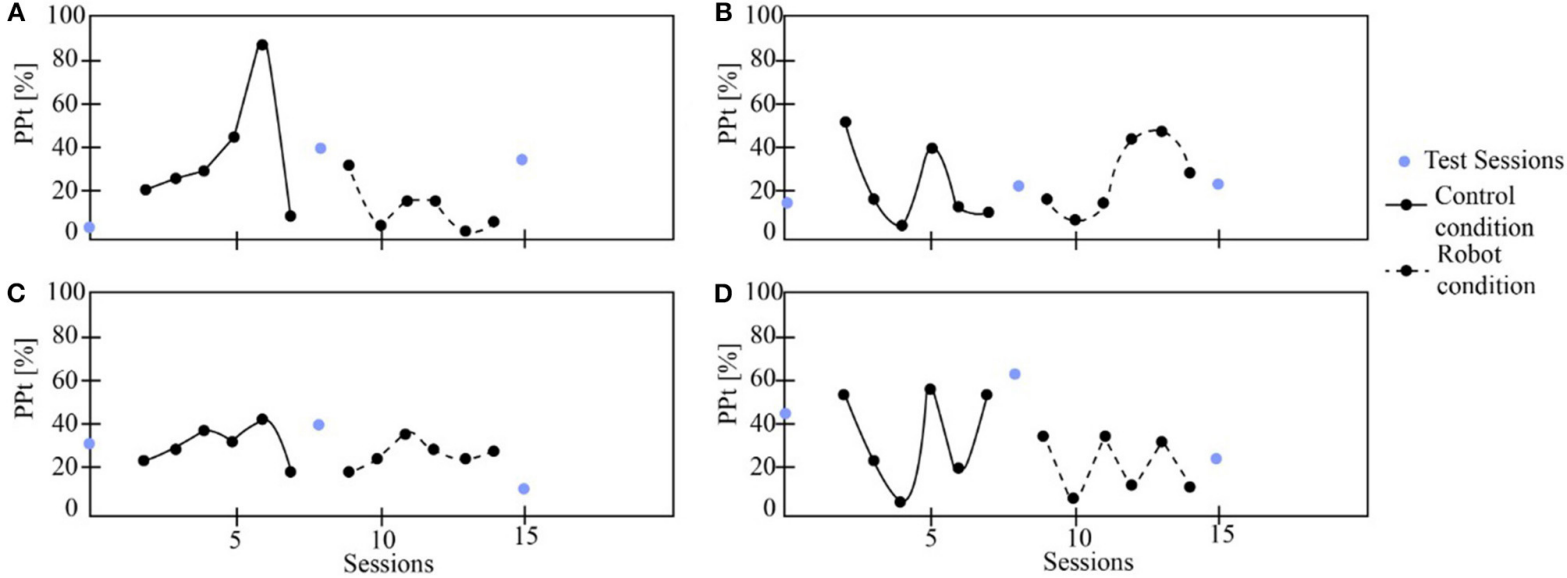
The patient's physiological progress starts with the control condition. **(A)** Cervical *PP*_*t*_ (sagittal plane), **(B)** Cervical *PP*_*t*_ (coronal plane), **(C)** Thoracic *PP*_*t*_ (sagittal plane), **(D)** Thoracic *PP*_*t*_ (coronal plane).

**Figure 7:**
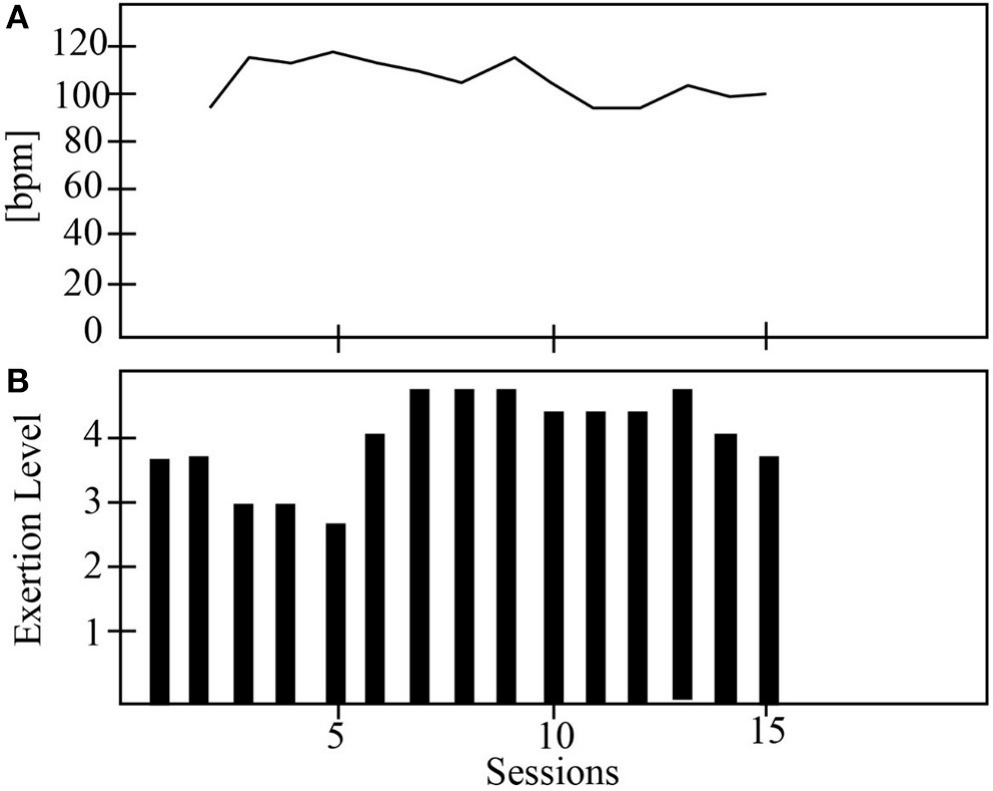
The physiological progress of a patient that starts with the control condition. **(A)** Heart rate and **(B)** Borg scale.

**Figure 8:**
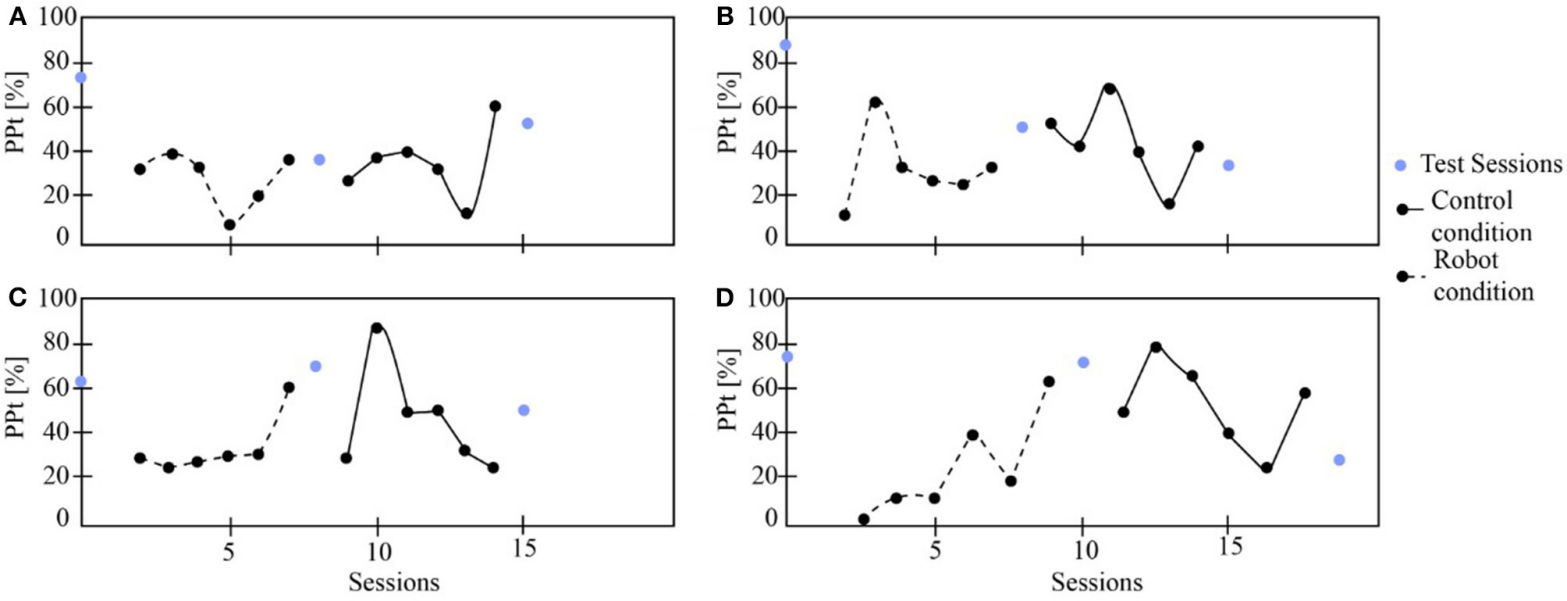
The patient's physiological progress who starts with the robot condition. **(A)** Cervical *PP*_*t*_ (sagittal plane), **(B)** Cervical *PP*_*t*_ (coronal plane), **(C)** Thoracic *PP*_*t*_ (sagittal plane), **(D)** Thoracic *PP*_*t*_ (coronal plane).

**Figure 9:**
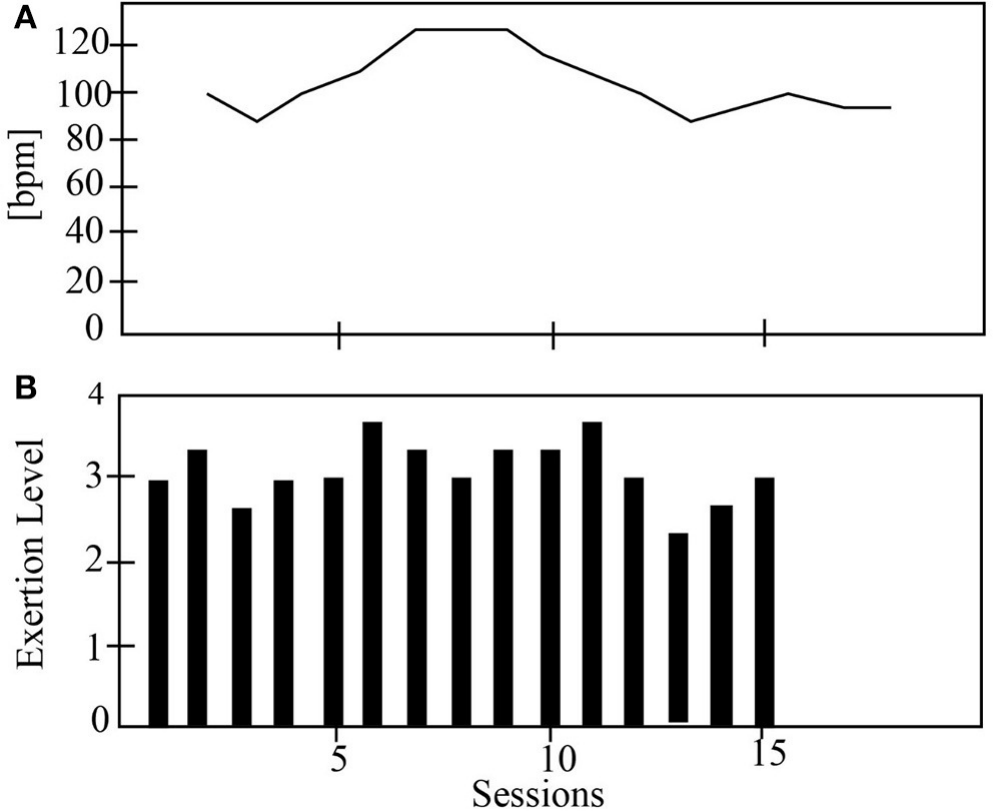
The physiological progress of a patient starts with the robot condition. **(A)** Heart rate and **(B)** Borg scale.

Figures 6–**9** show the patient's physiological progress regarding the cervical and thoracic posture, the heart rate, and the Borg scale. Figure 6 shows one patient's physiological parameter that starts the study with the control condition. In the cervical posture (Figures 6A,B), for both planes (sagittal and coronal) the percentage of *PP*_*t*_ decreases when the patient performs the session with the robot. The same result can be seen for the thoracic posture (Figures 6C,D). Moreover, the heart rate was maintained in a healthy range considering the exercise performed during the session. Also, the Borg scale was at low-perceived level (Figure 7).

On the other hand, Figure 8 presents one patient's physiological data who started the study with the robot. The cervical *PP*_*t*_ (sagittal and coronal planes) was lower with the social robot-assisted therapy (Figures 8A,B). An impressive result is that the patient tends to maintain the posture after the robot intervention (Figure 8A). This result could initially indicate that the patient learns how to control the cervical posture on the sagittal plane. This task corresponds to looking straight while performing the gait therapy with the Lokomat. In the case of thoracic posture (Figures 8C,D), it can be seen that the percentage of *PP*_*t*_ in this area was lower when using the robot. Finally, both the heart rate and the Borg scale were performed in healthy ranges (Figure 9).

**Figure 10:**
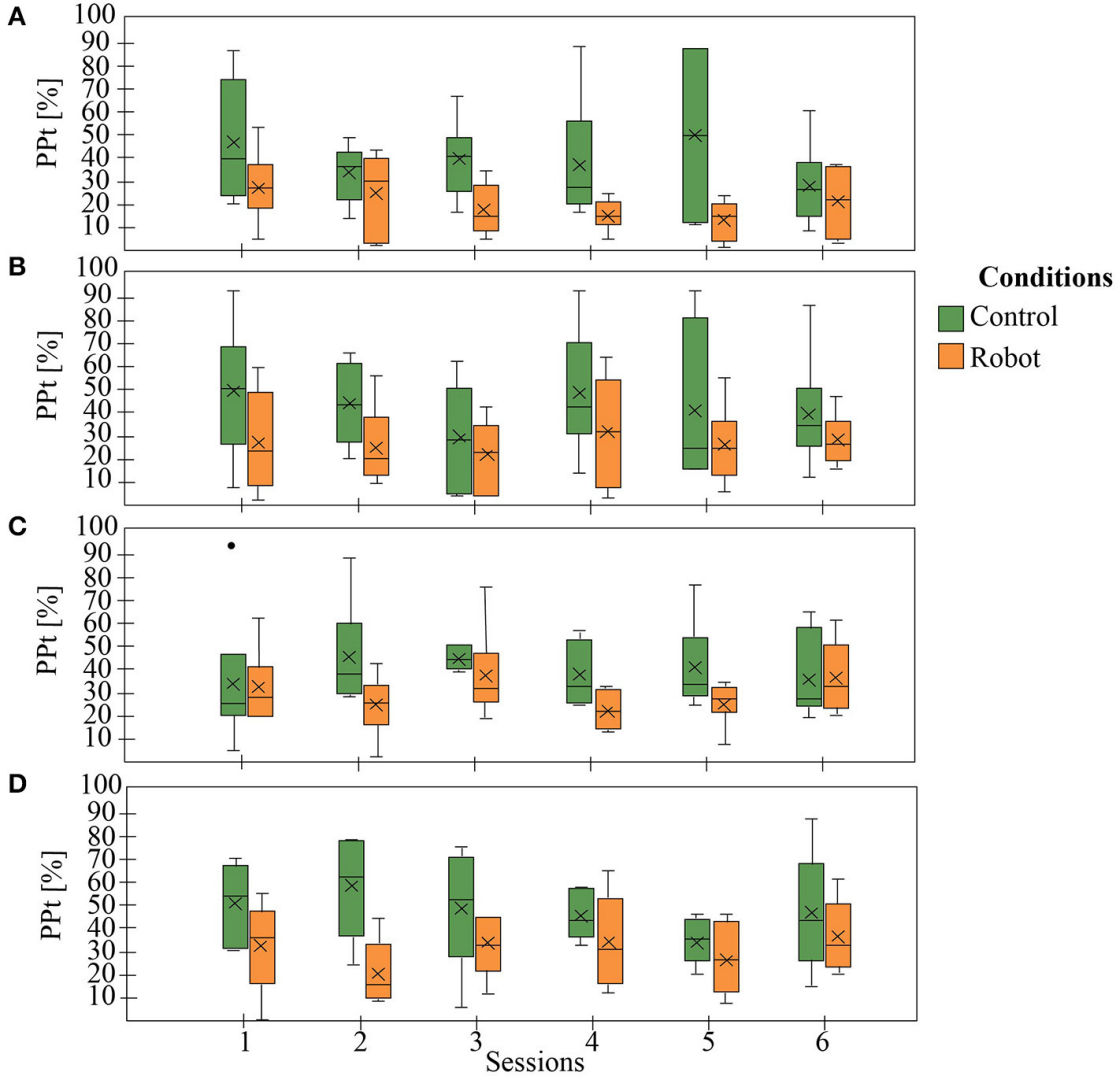
General patient's physiological progress. **(A)** Cervical *PP*_*t*_ (sagittal plane), **(B)** Cervical *PP*_*t*_ (coronal plane), **(C)** Thoracic *PP*_*t*_ (sagittal plane), **(D)** Thoracic *PP*_*t*_ (coronal plane).

Table 2 shows the *p*-values obtained after applied the Wilcoxon Signed-Rank test to the physiological data (i.e., *PP*_*t*_). There is a significant difference between the control and the robot condition regarding the *PP*_*t*_ for sagittal and coronal plane in both spinal areas. For instance, in the robot condition the percentages where the patients maintain an unhealthy posture are lower than the control condition (Figure 10). This outcome demonstrates the positive impact of the robot during the sessions, this effect could be related to the constant feedback provided to the patients and their willingness to achieve and maintain a healthy posture during the sessions. Furthermore, the heart rate and the Borg scale parameters, do not show differences between groups. This result can be due to the high dependence of the heart rate and the Borg scale of the patient's exercise during the therapy with Lokomat.

**Table 2:** Wilcoxon ranked signed test results.

**Measurement**	***p*-value**	***PP*_*t*_ Mean** **control condition ** **[*%*]**	***PP*_*t*_ SD** **Control condition** **[*%*]**	***PP*_*t*_ Mean** **Robot condition** **[*%*]**	***PP*_*t*_ SD** **Robot condition** **[*%*]**
Cervical *PP*_*t*_ (sagittal plane)	**0.01**	39.18	23.05	20.41	13.18
Cervical *PP*_*t*_ (coronal plane)	***p*****<0.01**	36.56	22.61	23.01	15.29
Thoracic *PP*_*t*_ (sagittal plane)	***p*****<0.01**	39.31	18.70	29.10	14.39
Thoracic *PP*_*t*_ (coronal plane)	***p*****<0.01**	46.9	19.71	30.8	17.88

*Robot and control conditions comparison for intra-subject analysis. Bold values are the p-values obtained after the Wilcoxon Signed-Rank test. They are bolding as they show significant differences between the conditions applied*.

**Figure 11:**
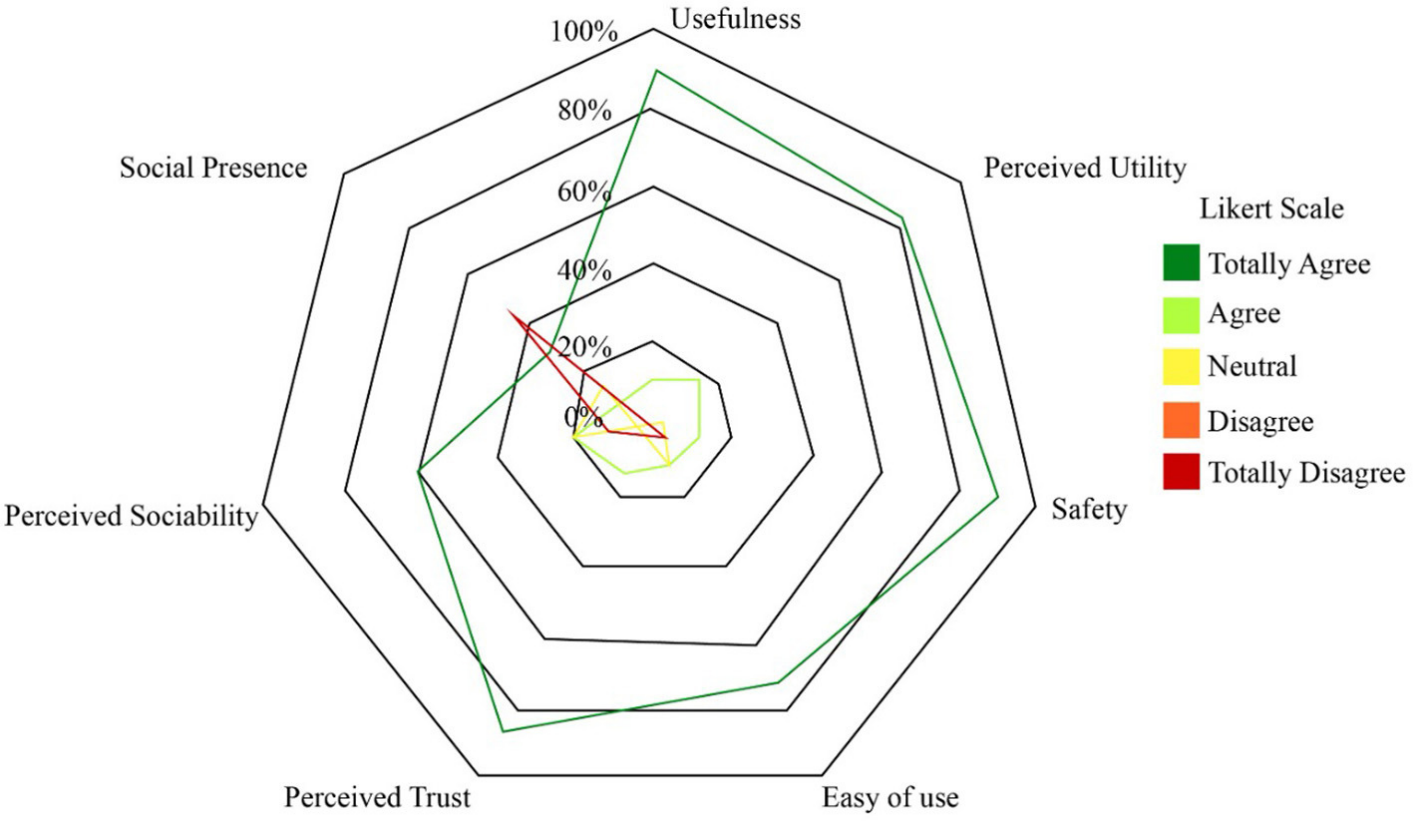
Chart of the percentage of the number of responses for each category. Social Presence (SP), Perceived Sociability (PS), Perceived Trust (PT), Ease of Use (EU), Safety (S), Perceived Utility (PU), and Usefulness (U).

The qualitative data analysis was performed to measure patient's interaction and attitudes toward the robot role during Lokomat therapy. Figure 11 shows the percentage on the Likert scale regarding each construct. It can be observed, that the patients have a positive perception of the robot in most of the constructs (U, PU, S, EU, and PT). In contrast, for the social presence (SP) construct a negative perception was elucidated by the participants.

**Figure 12:**
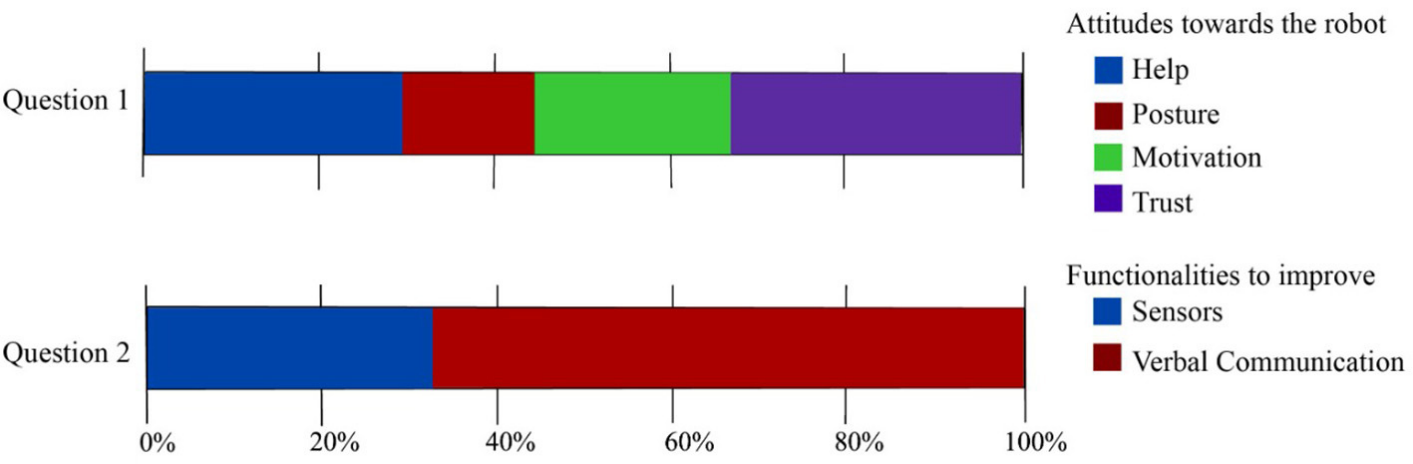
Results regarding the open questions of the UTAUT questionnaires. Attitudes toward the robot's role in neurorehabilitation and functionalities to improve were assessed.

Two open questions were analyzed using the frequency of the answers regarding the essential social robot's aspects (Figure 12). Question 1, reflects the clinicians' perceptions regarding the social robot. The answer elucidates the platform produces feelings of *help* (28.32%) and *trust* (36.47%) to the participants. The patients also use the words *posture* (15.42%) and *motivation* (21.46%) to describe the robot. Question 2 is focused on evaluating which factors could be improved in the therapy assisted by the robot; 68.21% of the patients' answer that the robot's dialogues could be less repetitive and 33.17% recommend inserting more *sensors* to improve reliability.

**Figure 13:**
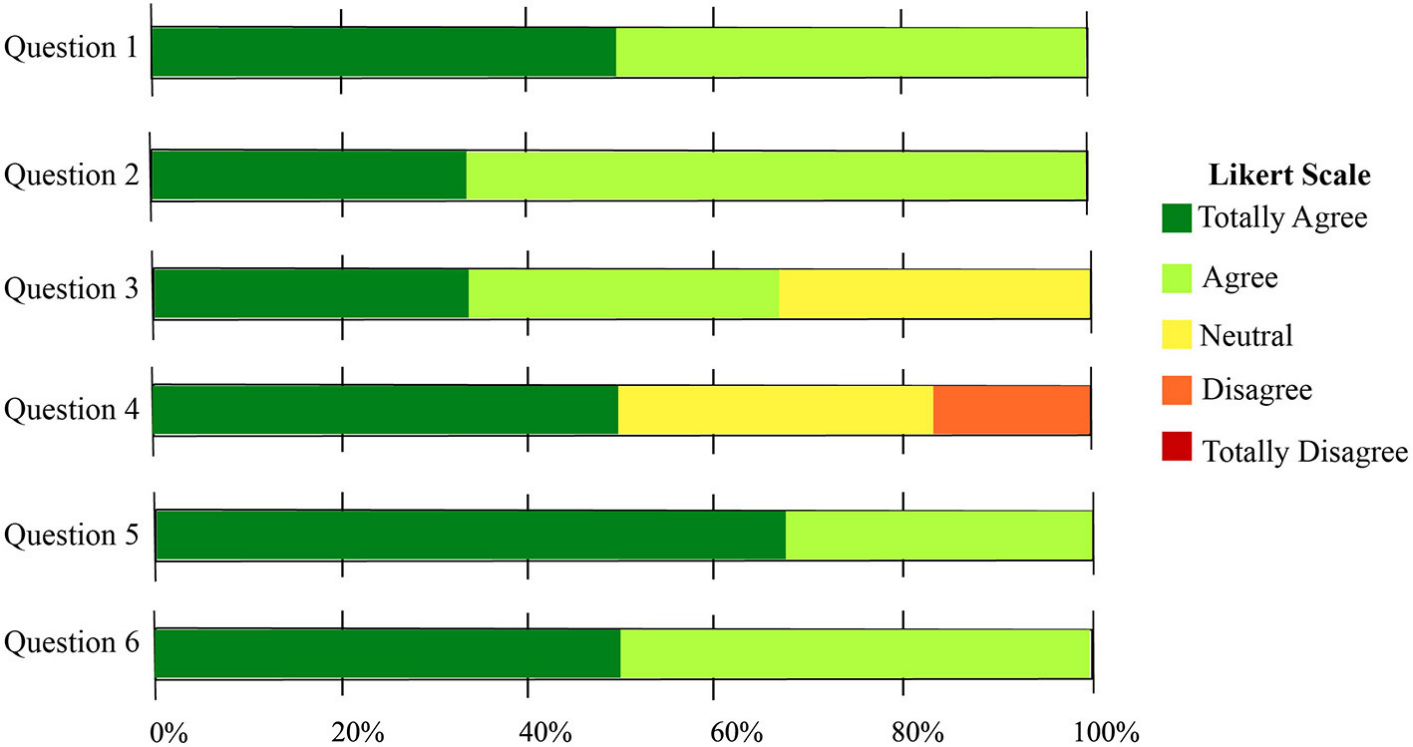
Results regarding the closed questions of the COVID related questionnaire. The usability of the robot in a rehabilitation scenario was assessed.

Furthermore, Figure 13 shows the results of the clinicians' perception regarding the robot's role in neurorehabilitation during the pandemic. In general, the healthcare personnel will *agree* (50%) and *totally agree* to use the robot during the pandemic, and recommend the robot to other colleagues to use the robot (Question 6). On the other hand, most clinicians agree with the fact that the robot can promote the physical distancing between the healthcare personnel and the patients. Within Question 4, a small percentage of clinicians answer that they disagree with the capability of the robot to support all of the tasks during the pandemic carried out in rehabilitation procedures. This result can be due to the limitations of the robot and highlighted in the open questions of the UTAUT questionnaire (Figure 12).

In the case of the open questions, the clinicians remarked on several advantages of the robot during the pandemic (e.g., “* During the pandemic using robots could promote the distancing, the visual and hearing feedback,” “ Continuous feedback and motivation,” “ It allows distancing, greater interaction of the patients, the robot does not condition the answers.'*). As disadvantages the health care commentaries were: “* There are limitations regarding some verbal feedback of the robot,”* and “* If the robot does not coordinate properly with the team's feedback, it can generate dispersion of attention, confusion in the orders of the therapist and the team.”* Finally, as additional features the therapists suggest to increase the robot's mobility in the scenario, and add the robot's behaviors at the end of the session to give some recommendations regarding the COVID-19 pandemic.

## 5. Discussion

This article presents a long-term study that involves ten patients who perform actively in Lokomat gait rehabilitation. A social robot supported these patients. The roles were to provide feedback and monitor the physiological progress of the patient. Two main variables were included in the study: (i) quantitative variables to measure the physiological progress, and (ii) qualitative variables to measure the interaction and patient's perception of the patients toward the robot.

The results show that the posture improves with the robot's assistance in the thoracic and cervical areas. Also, there is a statistical difference between the robot and the control condition. These results are very encouraging, as they show the robot's positive impact on the patient's physiological behavior. The feedback provided by the robot allows the patient to maintain a healthy posture and promote full gait rehabilitation. Moreover, the medical team also benefits from the robot's support, as the patient is continuously monitored and their ability to perform other tasks during the session increases. Within the study it was observed that the clinicians do not interfere with the robot's work and trust in the platform. Hence, in the COVID-19 pandemic, this tool could be handy as it allows the clinicians to complete the rehabilitation sustaining the social distancing with the patients, and decrease the contagion rate.

On the other hand, the system enables continuous monitoring of the patient. For instance, the heart rate is not measured in conventional therapies. With the system and the robot's interaction the clinicians could be warned by the robot and take action during the therapy if the patient has a high heart rate. Additionally, at the end of the rehabilitation, the clinicians could evaluate the patient progress, not only in the gait behavior but also in their cardiovascular functioning and the exertion perceived during each session. Through the questionnaire, the clinicians highlight that they trust in the system as a complementary tool in rehabilitation. Regarding the robot's role during the COVID-19 pandemic, the clinicians have a positive perception of the robot to use it as a tool to manage the rehabilitation procedures. Most of the healthcare personnel will use the robot during the pandemic, as they consider this tool can promote physical distancing and it is a secure device to carry out the healthcare protocol. Also, another encouraging result is that the clinicians will recommend the robot to other colleagues and institutions to support rehabilitation during the COVID-19 pandemic.

The qualitative results highlight the positive patient's perception and acceptance of the social robot. The patients perceived that the robot helped give feedback on the physiological parameters and maintain their healthy posture. Additionally, they considered that the system was very safe and secure as they were continuously monitored. Within the conventional Lokomat sessions, the cardiovascular response is not measured. In this case, the clinicians and patients consider this parameter fundamental to perform a safe therapy. In contrast, the patients have a neutral perception of the social presence and robot sociability. This result can be due to the repeatability of the dialogues and the robot's behaviors during the session. The patient's commentaries suggest that a fluid speech and conversation with the robot could improve the patient-robot interaction and sociability. This limitation could be enhanced by implementing strategies (e.g., face recognition and speech recognition) in subsequent studies. For example, in Libin and Libin (2004) the use of adaptive behaviors regarding the user personality increases motivation and quality of the interaction. Furthermore, the clinicians remark to add behaviors at the end of the session where the robot can make recommendations to the patients over the COVID-19 pandemic. For instance, washing hand protocols, correct use of the mask, among others.

Although the robot's sociability was perceived as lower, the patients highlight the platform's potential in Lokomat therapy. At the end of the sessions, most of the patients suggest using the robot with other patients, due to its reliability and help during the rehabilitation procedures. Also, some patients answer that the robot could enhance the health personnel tasks, and consequently, their trust in the sessions was higher.

## 6. Conclusions and Future Work

This paper presents the evaluation of a social robotic platform for neurorehabilitation with Lokomat. A total of 10 patients were evaluated during 15 sessions. The patients perform conventional and robot-assisted therapy starting the conditions randomly, to assess their performance in both scenarios.

Overall, the results evidence a positive effect of the social robot in the patient's physiological progress and interaction. The study's primary outcomes show that the patients improved their spinal posture (cervical and thoracic) when the social robot assisted them. The platform also allowed the on-line monitoring of patients' gait performance and cardiovascular functioning.

Regarding the perception, most of the patients highlight the platform's capability to aid their rehabilitation procedures and enhance the therapy for the patients and the clinicians. In contrast, they suggest that the sociability of the robot could increase using communicative and speech techniques. Thus, in future works a system that includes strategies to promote long-term interaction will be implemented. On the other hand, most of the assistive platforms as Lokomat are focused on assist the patients in a physiological way, however, the cognitive support it is essential to achieve a comprehensive procedure and adhere the patients to the treatment. In this way, SAR can be a potential tool to offer a cognitive approach and support clinicians in their tasks. These outcomes became more relevant with the COVID-19 pandemic, where clinicians need tools to assist patients in a safer manner; and the continuity of the rehabilitation is essential to maintain the patient's quality of life.

## Data Availability Statement

The raw data supporting the conclusions of this article will be made available by the authors, without undue reservation.

## Ethics Statement

The studies involving human participants were reviewed and approved by The ethics committee at Colombian School of Engineering. The patients/participants provided their written informed consent to participate in this study.

## Author Contributions

NC developed the social robotic platform and the UTAUT questionnaires. DR performed the clinical validation of the interface and process the study's data and including the statistical analysis. NC and DR led the manuscript writing. MM developed the experimental protocol. CC proposed and supervised the structure of the paper. MM and CC were involved in the revising and correcting the manuscript. All authors contributed to the article and approved the submitted version.

## Conflict of Interest

The authors declare that the research was conducted in the absence of any commercial or financial relationships that could be construed as a potential conflict of interest.

## Footnotes

^1^Some patients begun 3 weeks after the study beginning due the recruitment in the rehabilitation center.
