# Supplementary material for: Long-Term Social Human-Robot Interaction for Neurorehabilitation: Robots as a Tool to Support Gait Therapy in the Pandemic
Source: Front Neurorobot. 2021 Feb 23;15:612034. doi: 10.3389/fnbot.2021.612034 (PMC7959832; doi:10.3389/fnbot.2021.612034)
Supplement: Supplementary file 1 [file Table_1.pdf]

frontiers

Supplementary Material

1 SUPPLEMENTARY DATA

Table S1. Perception Questionnaire for Patients.

Construct	No.	Questions
U	1	I consider that using robots. It's an excellent tool to assist neurorehabilitation therapies.
	2	I consider that my interaction with the robot was comfortable.
	3	I enjoyed it when the robot gave me verbal encouragement when I did a good job.
	4	I'm satisfied with the work that the robot did.
	5	I consider that the robot adapts to my needs.
PU	1	I consider that the interaction with the robot was beneficial for my recovery.
	2	I consider that the role of the robot was necessary for therapy development.
	3	I think that the use of the robot helps me to compromise me to do a good job.
S	1	I feel safe at the therapies working with the robot.
	2	I consider it was easy to give information to the robot.
EU	1	I consider that the robot is easy to use.
	2	I consider that using the robot didn't affect the time of therapy sessions.
	3	I consider that the robot's instructions were clear.
PT	1	The robot made me confident.
	2	I did instruction the robot told me because I trusted him.
	3	I like using the robot during the therapies.
	4	It gave me confidence that the robot guides my therapy.
PS	1	I consider the robot a pleasant conversational partner.
	2	I find the robot pleasant to interact with.
	3	I feel the robot understands me.
	4	I think the robot is friendly.
SP	1	When interacting with the robot, I felt like I'm talking to a real person.
	2	It sometimes felt as if the robot was looking at me.
	3	I can imagine the robot to be a living creature.
	4	I often think the robot is not a real person.
	5	Sometimes the robot seems to have real feelings.

Table S2. COVID19-related questionnaire for Clinicians.

Question number	Description
1	The robot could be a useful tool to support neurorehabilitation with Lokomat during the COVID-19 Pandemic.
2	If I had the posibility, I would use the social robot as a platform in neurorehabilitation with Lokomat during th COVID-19 Pandemic.
3	The robot can promote physical distancing in the neurorehabilitation with Lokomat.
4	I think the robot can support my tasks within the session during the pandemic.
5	I think the social robot can be a safe tool to continue neurorehabilitation with Lokomat during the COVID-19 pandemic.
6	I would recomment to other colleagues/institutions the use of social robotics as a tool in the management of the COVID-19 pandemic.
Open Question 1	Mention 3 advantages of using a social robot in neurorehabilitation with Lokomat during the COVID-19 pandemic.
Open Question 2	Mention 3 disadvantages of using a social robot in neurorehabilitaton with Lokomat during the COVID-19 pandemic.
Open Question 3	Mention3 features you would added to the robot in order to improve its role in neurorehabilitation with lokomat during COVID-19 pandemic
